# Low-Dose Dietary Fish Oil Improves RBC Deformability without Improving Post-Transfusion Recovery in Mice

**DOI:** 10.3390/nu15204456

**Published:** 2023-10-20

**Authors:** Christopher Y. Kim, Hannah J. Larsen, Steven L. Spitalnik, Eldad A. Hod, Richard O. Francis, Krystalyn E. Hudson, Dominique E. Gordy, Elizabeth F. Stone, Sandy Peltier, Pascal Amireault, Angelo D’Alessandro, James C. Zimring, Paul W. Buehler, Xiaoyun Fu, Tiffany Thomas

**Affiliations:** 1 Department of Pathology and Cell Biology, Columbia University Irving Medical Center, New York-Presbyterian Hospital, New York, NY 10032, USA; 2 Bloodworks Research Institute, Seattle, WA 98102, USA; 3 Biologie Intégrée du Globule Rouge, Institut National de la Santé et de la Recherche Médicale, Université Paris Cité et Université des Antilles, 75014 Paris, France; 4 Laboratory of Cellular and Molecular Mechanisms of Hematological Disorders and Therapeutic Implications, Institut Imagine, INSERM, Université Paris Cité, 75005 Paris, France; 5 Department of Biochemistry and Molecular Genetics, University of Colorado School of Medicine, Aurora, CO 80045, USA; 6 Department of Pathology, University of Virginia School of Medicine, Charlottesville, VA 22903, USA; 7 Department of Pathology, University of Maryland School of Medicine, Baltimore, MD 21201, USA; 8 Department of Medicine, University of Washington, Seattle, WA 98195, USA

**Keywords:** fish oil, erythrocytes, transfusion, RBC, supplement, deformability, oxidative stress, lipid peroxidation, storage lesion, ROS

## Abstract

Long-chain polyunsaturated fatty acids (LC-PUFAs) are important modulators of red blood cell (RBC) rheology. Dietary LC-PUFAs are readily incorporated into the RBC membrane, improving RBC deformability, fluidity, and hydration. Female C57BL/6J mice consumed diets containing increasing amounts of fish oil (FO) ad libitum for 8 weeks. RBC deformability, filterability, and post-transfusion recovery (PTR) were evaluated before and after cold storage. Lipidomics and lipid peroxidation markers were evaluated in fresh and stored RBCs. High-dose dietary FO (50%, 100%) was associated with a reduction in RBC quality (i.e., in vivo lifespan, deformability, lipid peroxidation) along with a reduced 24 h PTR after cold storage. Low-dose dietary FO (6.25–12.5%) improved the filterability of fresh RBCs and reduced the lipid peroxidation of cold-stored RBCs. Although low doses of FO improved RBC deformability and reduced oxidative stress, no improvement was observed for the PTR of stored RBCs. The improvement in RBC deformability observed with low-dose FO supplementation could potentially benefit endurance athletes and patients with conditions resulting from reduced perfusion, such as peripheral vascular disease.

## 1. Introduction

Red blood cells (RBCs) perform a critical role in delivering oxygen to metabolically active tissues. Indeed, RBC number and hemoglobin content are primary determinants of exercise performance in endurance sports (e.g., running, cycling) [1]. Additionally, RBCs are the most transfused blood product, with >10 million units transfused annually in the United States [2]. Although RBCs are commonly regarded as a uniform blood product, each RBC unit is unique, as it comes from an individual donor, undergoes several manufacturing steps, and can be cold-stored for up to 42 days [3,4]. During storage, RBCs experience a series of changes termed the “storage lesion.” Most notably, these include a loss of surface area through vesiculation; decreased deformability, ATP, and pH; and increased phosphatidylserine externalization and lipid peroxidation [5,6,7,8,9,10,11]. Together, these deleterious effects are associated with poorer post-transfusion perfusion and function as compared to fresh RBCs. Given the critical need for optimally functioning endogenous RBCs in elite athletes and transfused RBCs in patients recovering from acute hemorrhage, understanding dietary factors influencing RBC quality is of particular interest.

RBC rheology is modulated by changing the fatty acid composition of the RBC phospholipid membrane. Specifically, increasing the proportion of long-chain polyunsaturated fatty acids (LC-PUFAs) relative to less saturated or saturated fatty acids increases membrane fluidity and deformability [12]. The fatty acid composition of RBC phospholipids can be modulated by altering the fatty acid profile in the diet; relative to plasma lipoproteins, which reflect recent dietary intake, RBC membranes reflect long-term dietary fatty acid intake, with the time to peak enrichment corresponding to the RBC lifespan (~110 days in humans) [13,14]. Fish oil (FO), composed of ω-3 fatty acids including docosahexaenoic acid (DHA), docosapentaenoic acid (DPA), and eicosapentaenoic acid (EPA), may offer many health benefits and is a commonly used nutritional supplement [15]. For example, the American Heart Association’s dietary guidelines recommend a daily dose of ~500 mg of FO to reduce the risk of death from coronary heart disease (CHD) in healthy individuals, whereas a daily FO dose of 1000 mg is recommended for individuals with CHD [16,17]. Dietary ω-3 LC-PUFAs rapidly integrate into RBC membranes, with incorporation half-lives in humans of 28.1 and 38.5 days for EPA and DPA, respectively, ref. [18]; by displacing less saturated fatty acids, membrane fluidity and RBC deformability both increase [12].

However, increasing lipid membrane desaturation may not be benign. For example, replacing saturated fatty acids with LC-PUFAs increases susceptibility to oxidative stress via reactive oxygen species (ROS). Thus, ROS react with LC-PUFAs by abstracting a hydrogen atom from the bis-allylic position, leaving an unpaired electron, thereby forming a fatty acid radical. Further reaction with oxygen produces peroxyl radicals, which can abstract hydrogen from neighboring LC-PUFAs during the propagation step, thereby exponentially expanding lipid peroxidation. Additionally, lipid peroxidation byproducts, such as highly reactive 4-hydroxynonenal (4-HNE), can also damage lipid membranes and cellular proteins by forming irreversible adducts [19,20,21].

We previously showed a causal relationship between lipid peroxidation and the storage lesion [22]. Although, at lower doses, FO has clinical benefits and DHA has antioxidant-like properties, large increases in membrane desaturation from high amounts of FO consumption could accelerate rates of lipid peroxidation, eliminating the benefits derived from improved membrane fluidity [23,24]. The current study examined whether FO supplementation could improve RBC quality before (i.e., fresh) and after cold storage. RBC quality measures were lifespan in vivo, deformability, filterability, and PTR.

## 2. Materials and Methods

### 2.1. Chemicals and Standards

FO diets were obtained from Envigo (Indianapolis, IN, USA). EZ-Link™ Sulfo-NHS-LC-Biotin (21335, biotinylating reagent) and APC-streptavidin were purchased from Thermo Fisher Scientific (349024, Waltham, MA, USA) and BD Biosciences (Franklin Lakes, NJ, USA), respectively. Phosphate buffered saline, 1× (PBS; 21-040-CM) was purchased from Corning (Corning, NY, USA). Leukoreduction filters (AP-4952) were purchased from Pall (Port Washington, NY, USA). Fatty acid methyl esters (FAME) standard mixture (FAME 25) was purchased from USP (1269119; Rockville, MD, USA), while methyl esters of arachidonic acid (AA, 2566-89-4), DHA (2566-90-7), DPA (108698-02-8), EPA (2734-47-6), and nonadecanoate (C19:0, 1731-94-8), as well as malondialdehyde (MDA, 100683-54-3), were purchased from Sigma (St. Louis, MO, USA). Malondialdehyde-D2 (MDA-D2, D-6469) was purchased from CDN Isotopes (Pointe-Claire, QC, Canada). Heptane (34873), hexane (34859), pentane (34956), methanol (34860), and hydrochloric acid in methanol (90964) were purchased from Sigma. Liquid chromatography-mass spectrometry (LC-MS) grade water was purchased from Supelco (Bellefonte, PA, USA, WX0001). Chloroform was purchased from J.T. Baker (67-66-3, Phillipsburg, NJ, USA).

### 2.2. Animal Experiments

All mouse experiments were approved by the Columbia University Irving Medical Center International Animal Care and Use Committee (AABH6563). C57BL/6J (Jackson Laboratory) mice were fed diets ad libitum, containing 0% FO (TD.180184), 6.25% FO (TD.180185), 12.5% FO (TD.180186), 25% FO (TD.180187), 50% FO (TD.180188), or 100% FO (TD.170504) as their lipid source, which constituted 30% of total caloric intake. After 1-week of acclimation, 10-week-old female C57BL/6J mice were randomly assigned to 1 of 6 different diets containing defined amounts of FO for 8 weeks (n = 15 mice/group). This timeframe corresponds to the approximate murine RBC lifespan [25].

Mice from the initial pilot analysis were fed custom-purified diets ad libitum, with the sole source of fat coming from: milkfat (TD.170501), olive oil (TD.170503), safflower oil (TD.170503), canola oil, or menhaden (fish) oil (TD.170504).

The AIN-93G modified FO diets are casein-based (fortified with L-cystine), with 17%, 30%, and 53% of the calories coming from protein, fat, and carbohydrate, respectively. The fat sources in each diet were provided by Envigo (Table 1). All diets contain Mineral mix AIN-93G-MX (35.0 g/kg), Vitamin Mix AIN-93-VX (10.0 g/kg), choline biterate (2.5 g/kg), and tert-butylhydroquinone (0.027 g/kg).

Mice were on a 12:12 h light/dark cycle. Mouse and food weights were recorded twice a week. Female C57BL/6J mice ubiquitously expressing GFP (C57BL/6J-TG UBC-GFP; Jackson Labs) were used as internal loading controls in PTR experiments. No sex differences in response to FO treatment were anticipated; thus, female mice were used as RBC donors and recipients.

### 2.3. In Vivo RBC Lifespan

At the start of the fifth week of feeding, RBCs were biotinylated in vivo (1 mg/300 μL in PBS injected into the retro-orbital sinus) [25]. Blood (1 μL) was collected from the tail vein after 1, 3, 7, 17, and 21 days and incubated with APC-streptavidin (1:100 *v*/*v* in PBS) for 40 min at room temperature. Samples were washed and resuspended in 1 mL of PBS and evaluated by flow cytometry (see below). The inferred RBC lifespan was determined by plotting the percentage of biotin-positive RBCs relative to the total number of RBCs. Flow data from days 1–21 (i.e., 5–8 weeks on diet) were analyzed by simple linear regression with the regression line extrapolated to the x-intercept to estimate the in vivo RBC lifespan for each diet group; the slope of the line represented the fractional RBC removal rate.

### 2.4. Blood Collection and Storage

On the day prior to blood collection, all RBCs were re-biotinylated in vivo, as above. Following cervical dislocation, blood was collected aseptically via cardiac puncture, pooled by diet, and CPDA-1 added (14% by volume). Leukocytes were removed via filtration (Pall Acrodisc; AP4952) using passive gravitational flow; the resultant blood was spun (1500× *g* for 10 min), packed to 60% hematocrit, and then stored for 12 days at 4 °C in 5.0 mL microcentrifuge tubes. Blood storage times were selected to closely mirror the FDA minimum requirement for 24 h PTR (i.e., >75%) for female C57BL/6J mice [26]. Blood units were prepared at Columbia University Irving Medical Center in New York City, NY. Fresh aliquots of these units (100 μL) were sent by international shipping (2-day transport) to Paris, France; for deformability (LORRCA) and filterability (microsphiltration) experiments. Temperature (4 °C) was maintained during transportation using cold packs.

### 2.5. 24-h PTR

On the morning of PTR experiments, a blood unit was prepared from UBC-GFP mice, as above, with a final hematocrit of 60%. Fresh (1-day stored) and stored blood units were spiked 1:5 with fresh UBC-GFP blood units as a loading control. The final hematocrit of these units was 60%. Aliquots (200 μL) from these spiked blood units were transfused via the retro-orbital venous sinus into 10-week-old female mice consuming a chow diet (n = 10/group). Blood samples were collected via tail puncture (1 μL in 1 mL PBS) at 5 min, 120 min, and 24 h post-transfusion and processed using the same workflow described for analyzing in vivo RBC lifespan. PTR was measured using flow cytometry (see below). PTR was calculated as the ratio of biotin-positive RBCs to GFP-positive RBCs circulating 24 h-post transfusion relative to the same ratio in the spiked blood unit.

### 2.6. Flow Cytometry

Flow cytometry used an Attune NxT with the No-Wash No-Lyse Filter kit (Thermo Fisher Scientific; USA) to detect RBCs. Aliquots (200 μL) of RBCs (1 μL/1 mL PBS) were acquired at a flow rate of 100 μL/min; 100,000 events were collected. The same acquisition settings were used to measure RBC lifespan and PTR.

### 2.7. Analysis of FAMEs by Gas Chromatography-Mass Spectrometry (GC-MS)

Dietary and RBC lipids were extracted using a modified Folch method. For diet analysis, two pellets of each diet (~3 g) were pulverized with a mortar and pestle. In a 1.5 mL microcentrifuge tube, 600 μL of 2:1 chloroform/methanol, 100 μL of liquid chromatography-mass spectrophotometry (LC-MS) grade water, and 20 μL of C19:0 methyl ester were added to 20 μL of RBCs or 50 mg of diet, vortexed for 10 min (room temperature), then centrifuged at 14,000× *g* for 10 min (4 °C). The supernatant and protein disc were discarded, and the bottom lipid layer was transferred to a 13 × 100 mm Pyrex screw cap tube with a silicone insert. Lipids were evaporated to dryness under nitrogen gas. The dried samples were transesterified to FAMEs with 100 μL of methanolic-HCL (100 °C for 1 h).

The FAMEs were extracted with hexane/water (2:1). The organic layer was transferred to a new tube, dried with sodium sulfate (~1 g), decanted into a new tube, and dried under nitrogen gas. The sample was solubilized in 500 μL of heptane and transferred to a 2 mL GC vial.

FAMEs were separated by a DB-FATWAX UI column (30 m, 250 μM diameter, 0.25 μM film thickness, Agilent Technologies, G3903-63008) using a 5975c GC/MS (Agilent Technologies, Santa Clara, CA, USA). One uL of the sample was injected (pulsed splitless) into the GC with the following settings: inlet temperature 250 °C; flow rate 1.0 mL/min; transfer line 280 °C; oven program mode was 80 °C, held for 1.5 min, then ramped to 240 °C (3 °C/min) and held for 7 min. FAMEs were quantified using selected ion monitoring (SIM) of *m*/*z* 55, 67, 69, 74, and 79 and GC-MS Agilent software version 07.01.

### 2.8. Analysis of MDA by GC-MS

After thawing a frozen aliquot (−80 °C) of each blood unit (25 μL) at room temperature, 25 μL of MDA-d2 (10 μg/mL in methanol), 100 μL of 2,4-dinitrophenylhydrazine (DNPH, 15.7 M in 2 M HCl, Sigma, D199303), and 200 μL of LC-MS grade water were added and mixed for 15 min. After 2 mL of pentane were added and the solution mixed for 15 min, the organic layer was transferred to a new vial, dried with anhydrous sodium sulfate, decanted to a new tube, and evaporated under nitrogen (60 °C). The sample was resolubilized with 100 μL of chloroform and transferred to a fixed-insert GC vial.

MDA was separated on an HP-5MS column (25 m, 200 μM diameter, 0.5 M film thickness, Agilent, 19091S-433) using a 5975c GC/MS. The sample (1 μL) was injected (pulsed splitless) into the GC with the following settings: inlet temp 250 °C; flow rate 1.0 mL/min; transfer line 2800 °C; oven program mode was 80 °C, held for 1 min, then ramped to 280 °C (7.5 °C/min), subsequently ramped to 280 °C (20 °C /min), and then held for 3 min. Quantification of MDA was carried out using SIM modes of *m*/*z* 158 and 160 for the endogenous molecule and the internal standard, respectively, with a 5-point calibration curve and GC-MS Agilent Quantitative software version 07.01.

### 2.9. RBC Deformability

RBC elongation index (EI) was measured by ektacytometry using a laser-assisted optical rotational red cell analyzer (LORRCA, RR Mechatronics, Zwaag, The Netherlands) over a shear stress range of 0.3–30 Pa, as described [27]. EI was defined as the ratio of the difference between the two axes of the ellipsoidal diffraction pattern and the sum of these two axes. Controls were fresh C57BL/6 RBCs. Deformability was performed on fresh (i.e., 2-day stored) and stored (12-day stored) samples.

RBC filterability was measured by microsphiltration. Microsphiltration plates were prepared as described previously [28]. Briefly, RBC suspensions (1% Hct in Krebs albumin 0.5%) were mixtures of 5% nonstained test or control RBCs and 95% CFSE-stained diluent RBC that were filtered through a microsphere layer. The proportion of non-stained test RBCs in the suspension was evaluated by flow cytometry (Canto II, BD Biosciences) and analyzed with computer software (FlowJo, V10, BD Biosciences). The retention rate was calculated using the formula Δ = {[(% of nonlabeled RBCs in the upstream sample) − (% of nonlabeled RBCs in the downstream sample)]/(% of nonlabeled in the upstream sample)} × 100.

Positive values, named retention, indicate that sampled RBCs were less deformable than diluent RBCs, whereas negative values, named enrichment, indicate the opposite. To validate each experiment, control samples were unstained diluent RBCs (negative control) and 0.8% glutaraldehyde-fixed RBCs (positive control).

### 2.10. Complete Blood Count (CBC)

Blood was collected from each donor mouse via cardiac puncture into microcentrifuge tubes containing EDTA. CBCs were performed on an Element HT5 CBC automated analyzer (Heska Corporation, Loveland, CO, USA).

### 2.11. Analysis of Free Long Chain-PUFAs (LC-PUFAs), Oxylipins, Phospholipids, and Lysophospholipids by LC-MS/MS

Free LC-PUFAs, oxylipins, and lysophospholipids (LPLs) in fresh and stored RBCs were analyzed by LC-MS/MS, as described [22,29,30,31]. Briefly, analytes were extracted with 80% methanol (vol/vol) with internal standards of: AA-d8, DHA-d5, EPA-d5, dihomo-γ-linolenic acid-d6 (DGLA-d6), α-linolenic acid-d14 (ALA-d14), linoleic acid-d4 (LA-d4), 15-hydroxyeicosatetraenoic acid-d8 (15-HETE-d8), 5-hydroxyeicosatetraenoic acid-d8 (5-HETE-d8), 12-hydroxyeicosatetraenoic acid-d8 (12-HETE-d8), 9-hydroxyoctadecadienoic acid-d4 (9-HODE-d4), 13-hydroxyoctadecadienoic acid-d4 (13-HODE-d4), 9,10-dihydroxyoctadecenoic acid-d4 (9,10-diHOME-d4), and 12,13-dihydroxyoctadecenoic acid-d4 (12,13-diHOME-d4) from Cayman Chemical (Ann Arbor, MI); 17:1 lysophosphatidylcholine (17:1 LPC), 17:1 lysophosphatidylethanolamine (17:1 LPE), 17:1 lysophosphatidylserine (17:1 LPS), and 17:1 lysophosphatidylinositol (17:1 LPI) from Avanti Polar Lipids (Alabaster, AL). LC-MS/MS was performed using a QTrap 6500 mass spectrometer (AB Sciex, Framingham, MA) coupled to an ultra-performance liquid chromatography (UHPLC) platform (Acquity I-Class, Waters, Milford, MA). Analytes were separated on a C18 column (Acquity HSS T3, 2.1 × 100 mm, 1.8 μm, Waters). Phospholipids were extracted using LC-MS-grade 2-propanol (final concentration 95% *v*/*v*) with Odd-chained LIPIDMIX from Avanti Polar Lipids. The extracted phospholipids were separated by LC using a C8 column (Acquity BEH C8, 1.7 mm, 2.1 3 100 mm; Waters). Analytes were detected using multiple reaction monitoring (MRM) in the negative ion mode and were quantified relative to their deuterium-labeled or 17:1 LPL or PL (17:0/14:1) analogs. Data were collected and processed using Analyst Version 1.6.2 and MultiQuant Version 2.1.1 (AB Sciex).

### 2.12. UHPLC-MS Metabolomics

Metabolomics analyses (e.g., acylcarnitine) were performed using a Vanquish UHPLC coupled online to a Q Exactive mass spectrometer (Thermo Fisher Scientific, Bremen, Germany) using 5, 15, and 17 min gradients, as described [32,33]. Data were analyzed using Maven (Princeton University, Princeton, NJ, USA) and Compound Discoverer 2.1 (Thermo Fisher Scientific).

### 2.13. Statistics

Statistical analyses were performed using Prism 9 (GraphPad; San Diego, CA, USA). Statistical tests for each experiment are defined in the figure legends. Flow cytometry data were analyzed using FlowJo 10 (BD Biosciences; Ashland, OR, USA); metabolomics analysis used MetaboAnalyst 5.0.

## 3. Results

### 3.1. Pilot PTR Study

**Figure 1:**
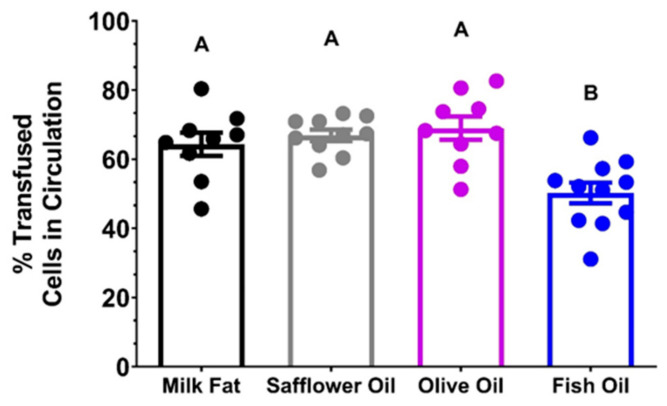
Pilot 24 h PTR Study of Stored RBCs (n = 9–11/group). Groups that do not share common letters statistically differed from one another by one-way ANOVA with Tukey’s multiple comparisons follow-up test (*p* < 0.05).

C57BL/6J mice were fed AIN-93M modified diets containing milkfat, olive oil, safflower oil, canola oil, and menhaden (fish) oil as their exclusive lipid source for 8 weeks. Aliquots from stored blood units were transfused into C57BL/6J recipients. The 24 h PTR was significantly reduced in the FO group relative to the milkfat, safflower oil, olive oil, and canola oil groups (Figure 1). Therefore, FO was used in the subsequent studies.

#### 3.1.1. Dietary Lipids Are Incorporated into the RBC Membrane in C57BL/6J Mice

**Figure 2:**
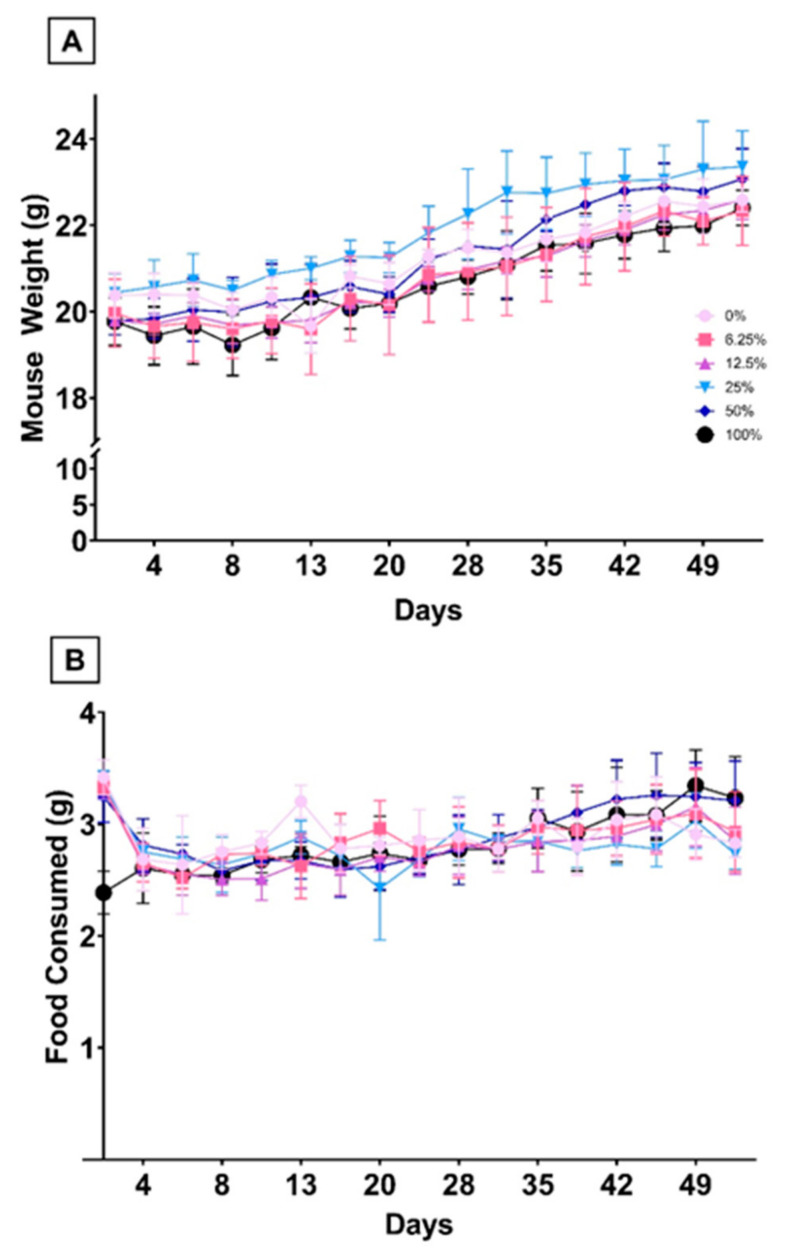
Food Intake and Body Weight. Animal weight (**A**) and food consumption (**B**) were measured twice per week throughout the study.

We examined the RBC fatty acid profiles of C57BL/6J mice fed AIN-93G modified diets differing in LC-PUFA content based on the percentage of FO in each diet. Diets were equally palatable, with no differences in food consumption or body weight seen in any group at any time point (Figure 2).

Lower-dose FO rodent diets are enriched in saturated and unsaturated medium- and long-chain fatty acids with no appreciable contribution of long-chain ω-3 and ω-6 LC-PUFAs (i.e., AA (20:4 ω-6), EPA (20:5 ω-3), DPA (22:5 ω-3), and DHA (22:6 ω-3),) whereas higher dose FO diets contain increasing amounts of LC-PUFAs and decreasing amounts of medium- and long-chain saturated and unsaturated fatty acids (Table 2A).

**Figure 3:**
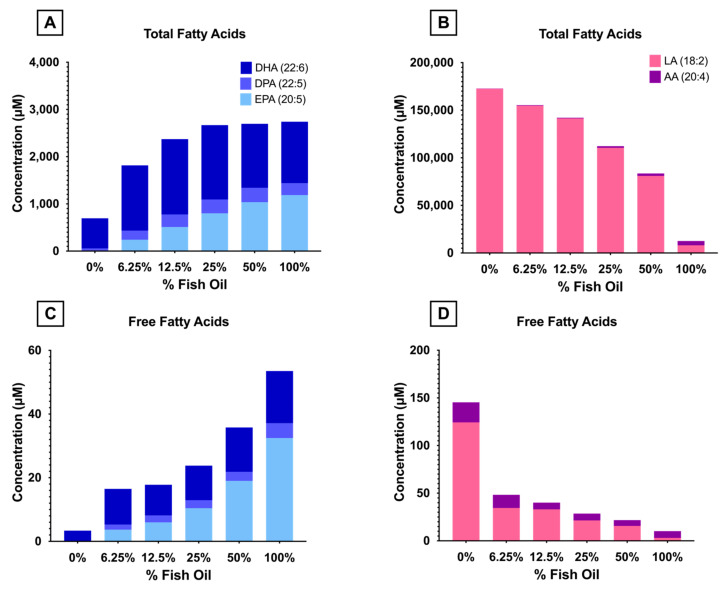
ω-3 and ω-6 total and free fatty acid concentrations in fresh RBCs were measured by GC-MS and LC-MS/MS. (**A**) Total ω-3 fatty acids. (**B**) Total ω-6 fatty acids. (**C**) Free ω-3 fatty acids. (**D**) Free ω-6 fatty acids.

The concentrations of total and free ω-3 and ω-6-LC-PUFAs in fresh RBCs (Figure 3) were quantified by GC-MS and LC-MS/MS, respectively. Consistent with increased levels of ω-3 PUFAs (EPA, DPA, and DHA) in the FO diets, these fatty acids were more enriched in the RBCs of mice consuming them. Both total and free EPA concentrations increased linearly with increased dietary FO, whereas DPA increased up to 50% FO and DHA increased up to 12.5% FO before reaching a plateau. Conversely, ω-6 PUFAs (LA, 18:2) in the diet decreased with increasing FO (Table 2A). RBC total and free concentrations of LA, along with AA (its metabolic elongation and desaturation product), decreased in a dose-dependent manner reflective of dietary LA intake (Figure 1).

#### 3.1.2. Dietary FO Was Not Associated with Differences in Hematological Parameters

No differences were seen in hematocrit, RBC count, hemoglobin, RBC distribution width, or mean corpuscular volume in the C57BL/6J mouse dietary groups (Table 3).

#### 3.1.3. Higher Doses of FO Were Associated with Reduced RBC In Vivo Lifespan

**Figure 4:**
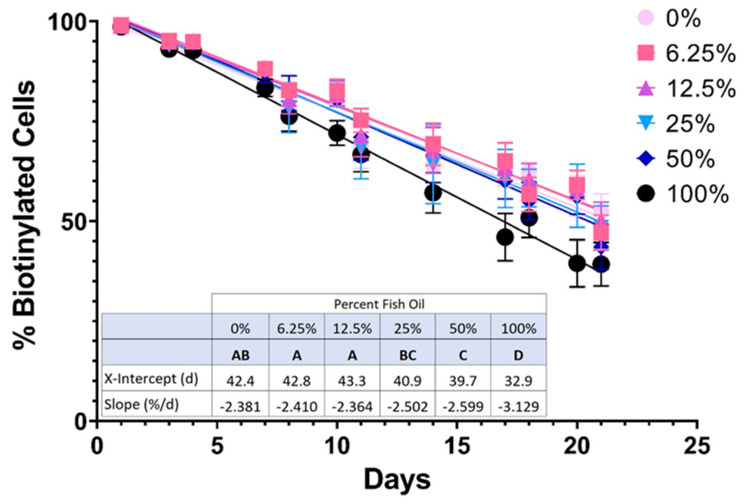
RBC lifespan in vivo is quantified by flow cytometry (n = 15 mice/group). Groups that do not share common letters differ statistically from each other by one-way ANOVA with Tukey’s multiple comparisons follow-up test (*p* < 0.05).

The inferred linear lifespans of RBCs in mice fed AIN-93M-modified diets were determined by biotinylating RBCs in vivo and then following the resulting clearance of labeled RBCs over the final 3 weeks of the feeding study (Figure 4). The slope (fractional RBC removal rate) and x-intercept (inferred linear RBC lifespan) of RBC survival data did not differ significantly in mice consuming moderate amounts of FO (6.25% and 12.5% FO) relative to the control group; however, RBC lifespan did decrease with further increases in FO dosage (i.e., 25%, 50%, and 100% FO).

#### 3.1.4. Lipid Peroxidation Increased in Mice Consuming High Dose FO

**Figure 5:**
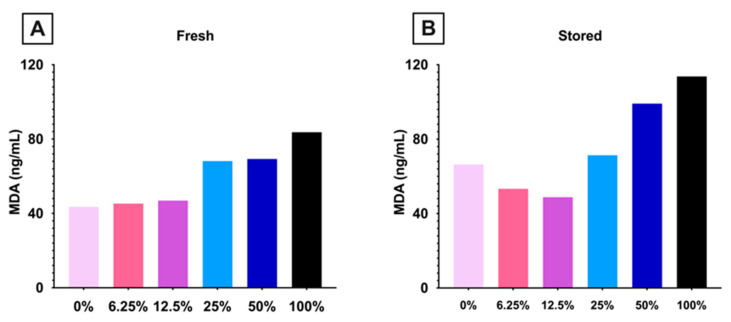
MDA was quantified in (**A**) fresh and (**B**) stored pooled blood units (15 mice/group; 1 analysis/blood unit). (0% FO: light pink; 6.25% FO: magenta; 12.5% FO: purple; 25% FO: light blue; 50% FO: royal blue; 100% FO: black).

To determine if dietary FO altered RBC lipid peroxidation, MDA, a relevant end-product, was measured in fresh and stored pooled blood units prepared (n = 15 mice/group). In fresh RBCs, MDA increased with increasing amounts of dietary FO (Figure 5A). In stored RBCs, MDA exhibited a biphasic response, decreasing in mice fed diets with 0–12.5% FO and then increasing in each subsequent FO dose (Figure 5B).

#### 3.1.5. RBC PL and LPL Levels Are Unaffected by Dietary FO

**Figure 6:**
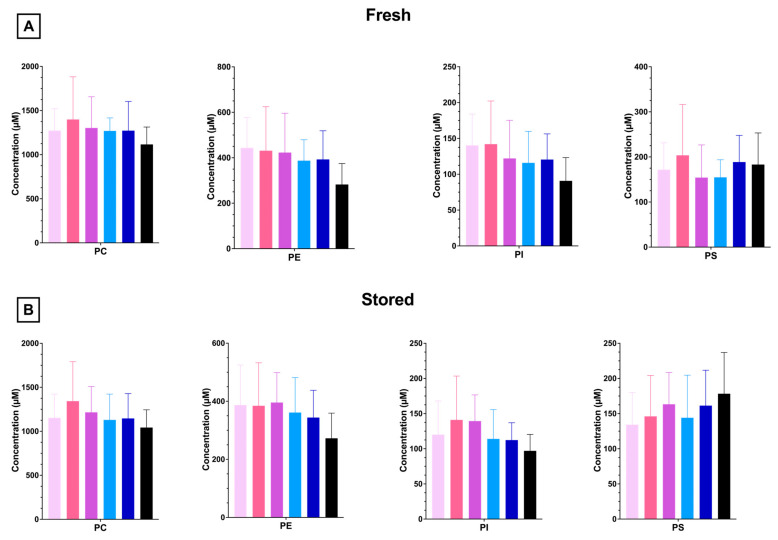
RBC PLs from 4 independent experimental replicates (n = 4) analyzed after 1 (**A**) or 12 (**B**) days of cold storage. Groups that do not share common letters differ statistically from each other by one-way ANOVA with Tukey’s multiple comparisons follow-up test (*p* < 0.05). (0% FO: light pink; 6.25% FO: magenta; 12.5% FO: purple; 25% FO: light blue; 50% FO: royal blue; 100% FO: black).

To understand whether increasing amounts of dietary FO modulated RBC phospholipid composition, phospholipidomics were performed with fresh and stored RBCs. The most abundant PL in mouse RBCs is phosphatidylcholine (PC), with decreasing amounts of phosphatidylethanolamine (PE), phosphatidylserine (PS), and phosphatidylinositol (PI). FO did not significantly alter the total RBC concentration or relative amounts of any PL species (Figure 6A). Cold storage (12 days) did not significantly alter the PL profile in any group relative to fresh units (Figure 6B and Supplementary Figure S1).

**Figure 7:**
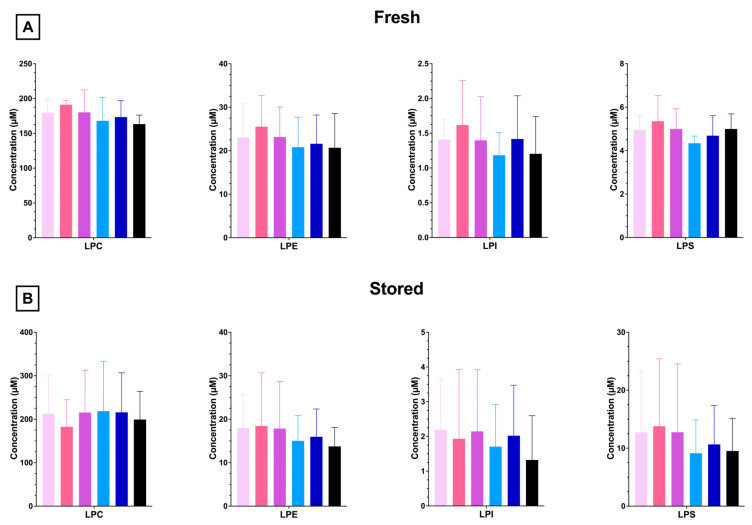
RBC LPLs from 4 independent experimental replicates (n = 4) were analyzed after 1 (**A**) or 12 (**B**) days of cold storage. Groups that do not share common letters differ statistically from each other by one-way ANOVA with Tukey’s multiple comparisons follow-up test (*p* < 0.05). (0% FO: light pink; 6.25% FO: magenta; 12.5% FO: purple; 25% FO: light blue; 50% FO: royal blue; 100% FO: black).

PLs can be enzymatically catabolized to LPLs by phospholipases (e.g., phospholipase A2). Similar to what was observed in the PL profile, lysophosphatidylcholine (LPC) is the most abundant LPL species, with a decreased abundance of LPE, LPS, and LPI (Figure 7A). No significant differences in LPL composition were observed with any dose of FO in fresh or stored blood units (Figure 7A,B). After cold storage, LPS and LPI levels were significantly elevated relative to fresh units, whereas no differences were observed for LPC or LPS (Supplementary Figure S2).

#### 3.1.6. Free PUFAs and Oxylipins

**Figure 8:**
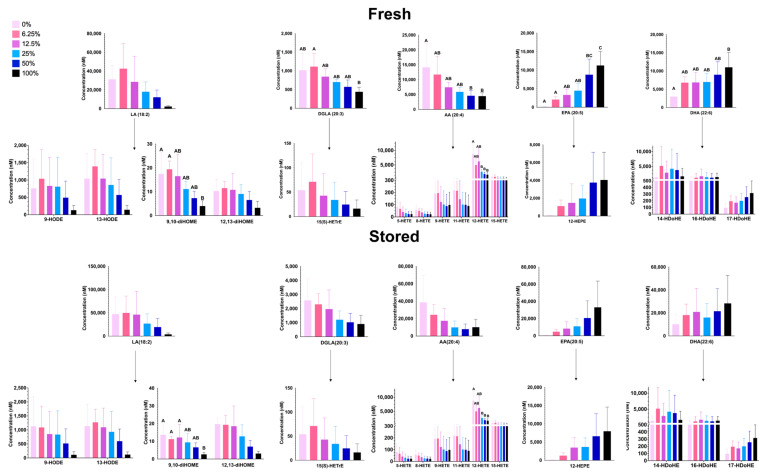
Free LC-PUFAs and metabolites of LC-PUFAs (i.e., oxylipins) in fresh (**A**) and stored (**B**) RBCs from 4 independent experimental replicates. Groups that do not share common letters differ statistically from each other by one-way ANOVA with Tukey’s multiple comparisons follow-up test (*p* < 0.05). (0% FO: light pink; 6.25% FO: magenta; 12.5% FO: purple; 25% FO: light blue; 50% FO: royal blue; 100% FO: black).

Following phospholipase cleavage of PLs, liberated free fatty acids can be spontaneously (i.e., peroxidation) or enzymatically metabolized to lipids with bioactive properties (i.e., oxylipins). Oxylipin levels reflected the levels of their respective free fatty acid precursors; that is, oxylipin levels derived from ω-3 PUFAs increased with increasing FO, and oxylipin levels derived from ω-6 PUFAs decreased with increased amounts of FO in both fresh and stored RBCs (Figure 8). Although free fatty acid levels increased after storage, the ratio of stored/fresh fatty acid concentrations did not reach statistical significance for most free fatty acids (Supplementary Figure S3). 

#### 3.1.7. High Dose FO Is Associated with Reduced Deformability and Filterability

**Figure 9:**
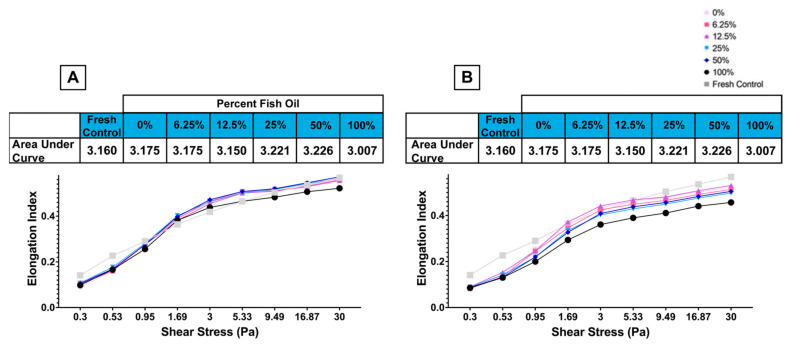
Deformability of (**A**) fresh (i.e., 2-day stored) and (**B**) stored RBCs at various shear stresses (representative example: 2 independent experimental replicates).

RBC elongation, a measure of deformability, was measured by LORRCA using a wide range of shear stresses representing physiological pressures found in the vasculature (1–2 Pa) and microvasculature (2–10 Pa), along with supraphysiological shear stress (>10 Pa) [34,35]. The 100% FO diet was associated with reduced deformability relative to other groups at shear stresses greater than 3 Pa with fresh (Figure 9A) and 0.95 Pa with stored (Figure 9B) RBCs. No differences were observed relative to fresh RBCs at other FO doses.

**Figure 10:**
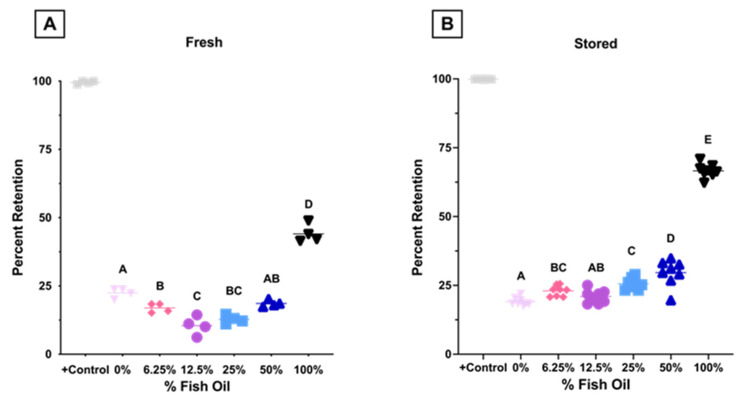
Microsphiltration was used to assess the deformability of (**A**) fresh (i.e., 2-day stored, n = 4/group) and (**B**) stored (n = 8/group) RBCs. Glutaraldehyde-treated RBCs are used as controls. Groups that do not share common letters differ statistically from each other by one-way ANOVA with Tukey’s multiple comparisons follow-up test (*p* < 0.05). (representative example: 2 independent experimental replicates). (0% FO: light pink; 6.25% FO: magenta; 12.5% FO: purple; 25% FO: light blue; 50% FO: royal blue; 100% FO: black).

Using an ex vivo splenic sequestration model (i.e., microsphiltration), where less deformable RBCs are retained within a bead matrix, fresh RBCs exhibited a biphasic response with increased filterability (i.e., more deformable) at 0–12.5% FO, before a subsequent decrease at 25–100% FO (Figure 10A). After 12 days of cold storage, RBCs exhibited a dose dependent decrease in filterability (i.e., decreased deformability) at FO doses above 12.5% (Figure 10B).

Taken together, these results show that RBCs from mice fed a high-dose FO diet (100% FO) were less deformable at physiological and supraphysiological shear stresses and had reduced filterability by microsphiltration before and after storage. Conversely, improved filterability was observed in mice fed lower-dose FO diets (6.25–25%) in fresh RBCs relative to the control.

#### 3.1.8. High Dose FO Diets Were Associated with Decreased 24 h PTR after Storage

**Figure 11:**
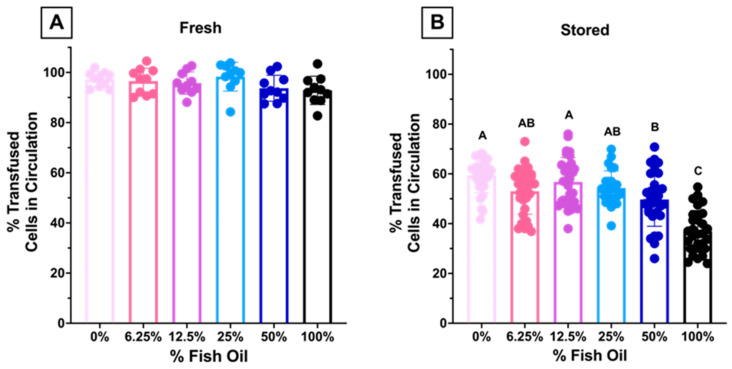
Approximately 24-PTR of (**A**) fresh (n = 10/group) and (**B**) 12-day stored RBCs (n = 30/group). Groups that do not share common letters differ statistically from each other by one-way ANOVA with Tukey’s multiple comparisons follow-up test (*p* < 0.05). (0% FO: light pink; 6.25% FO: magenta; 12.5% FO: purple; 25% FO: light blue; 50% FO: royal blue; 100% FO: black).

Aliquots from fresh and stored blood units were transfused into C57BL/6J recipients. There were no differences in 24 h PTR when fresh RBCs were transfused into recipients (Figure 11A). However, after 12 days of cold storage, there were significant decreases in PTR in recipients transfused with RBCs from donors fed 50% and 100% dietary FO, as compared to lower-dose FO or controls (Figure 11B).

#### 3.1.9. Stored RBC 24 h PTR Correlates with Stored RBC Deformability and Filterability

**Figure 12:**
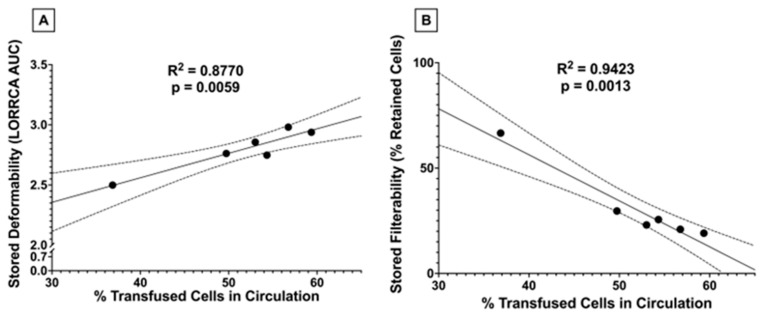
Correlations between stored 24 h PTR and (**A**) stored deformability and (**B**) stored filterability. R2, along with the significance of the linear relationship, were assessed by simple linear regression (n = 6). Dotted lines represent the 95% confidence interval.

After observing a significant decline in PTR when transfusing RBCs from donors receiving higher doses of dietary FO, we assessed whether there were associations between stored PTR and RBC deformability and filterability. Significant correlations were observed between PTR and stored filterability and with stored deformability (Figure 12).

#### 3.1.10. RBC Lifespan In Vivo Correlates with Fresh Filterability, but Not with Fresh Deformability

**Figure 13:**
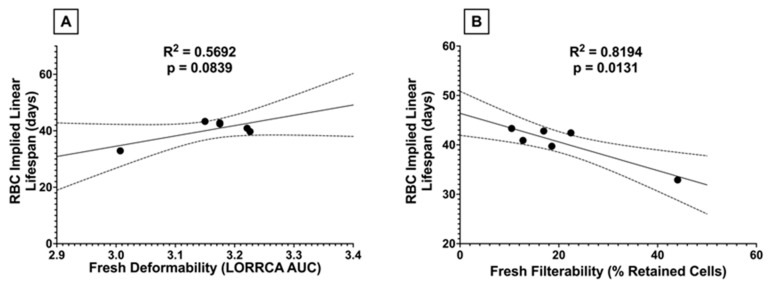
Correlations between RBC implied linear lifespan in vivo and (**A**) fresh deformability or (**B**) fresh filterability were assessed by simple linear regression. The dotted lines represent the 95% confidence intervals.

After observing a relationship between RBC deformability and PTR, we asked whether dietary FO-driven changes in deformability were associated with differences in RBC lifespan in vivo. Although a significant correlation was found between fresh filterability and RBC lifespan in vivo (Figure 13B), the relationship between fresh RBC deformability and lifespan in vivo did not reach statistical significance (*p* = 0.08; Figure 13A).

#### 3.1.11. Carnitine Levels Decrease with Higher Dose FO Diets

**Figure 14:**
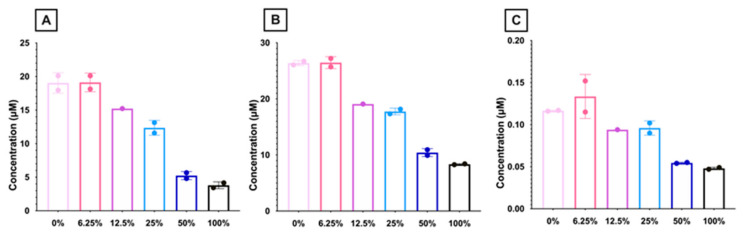
RBC levels of (**A**) free carnitine, (**B**) esterified carnitine, and (**C**) long-chain acyl carnitines. Long-chain acyl carnitine was calculated as the sum of C16, C16:1, C18, C18:1, and C18:2-carnitine (n = 1–2 replicates/group). (0% FO: light pink; 6.25% FO: magenta; 12.5% FO: purple; 25% FO: light blue; 50% FO: royal blue; 100% FO: black).

Metabolomics analysis of RBCs from mice fed different FO diets was performed to identify potential mechanisms explaining the observed differences in RBC quality. To this end, free and esterified carnitine levels were quantified (Figure 14). Free (Figure 14A) and esterified (Figure 14B) carnitine levels decreased with increasing amounts of dietary FO. Long-chain acyl carnitines, which are a reservoir for acyl-CoA for LPL re-acylation, also decreased with high-dose FO.

## 4. Discussion

These results support the axiom, “you are what you eat”. Thus, the RBC fatty acid composition of mice consuming AIN-93M-modified diets with increasing amounts of FO was directly proportional to the composition of the different diets. The RBC fatty acid profile is an established marker of long-term (~110 days) dietary fatty acid intake [13]. Dietary lipids are incorporated into the RBC membrane via direct exchange with plasma lipoproteins in circulation as well as during erythropoiesis in the bone marrow. RBC-PL exchange with lipoproteins is rapid, with observable changes in lipid composition after several days [13,36]. However, RBC PL asymmetry would theoretically result in a more rapid and complete exchange of PL species on the outer leaflet (e.g., phosphatidylcholine) relative to PL species primarily located within the inner leaflet (i.e., phosphatidylserine, phosphatidylethanolamine, and phosphatidylinositol). When Trappist monks were given FO supplements daily for 6 months, the time to maximal RBC enrichment approximately correlated with RBC lifespan in vivo (i.e., ~16 weeks) [18]. Further, peak enrichment with ω-3 LC-PUFAs was dose-dependent.

Pilot studies in mice evaluating various dietary fatty acid profiles commonly observed in human diets showed no differences in PTR when the lipids were derived from sources enriched in saturated fat (milk fat), monounsaturated fat (olive oil), or ω-6 LC-PUFAs (safflower oil; Figure 1). However, a diet with 30% of calories derived from FO as the sole lipid source significantly reduced the 24 h PTR of 12-day cold-stored RBCs. We hypothesized that the reduced PTR was attributable to increased lipid peroxidation due to increased levels of desaturation in the RBC PL membrane. In addition, we hypothesized that lower doses of FO could improve RBC quality.

After feeding mice AIN-93M-modified diets with dose-increasing levels of FO for 8 weeks, the RBC fatty acid profile reflected dietary intake, such that higher doses of dietary FO proportionally increased levels of these same fatty acids in the RBC membrane (Table 2, Figure 3). Mice were fed diets containing pharmacologically relevant levels of FO (6.25% and 12.5% FO correspond to 4 and 8 g of FO for a human consuming 2000 kcal/d) and levels not commonly observed in normal diets or through pharmacological supplementation (25%, 50%, and 100% corresponding to 17 g, 33 g, and 67 g of FO). Menhaden oil, like most Fos, has a relatively small percentage of ω-3 LC-PUFAs relative to saturated and less saturated fatty acids. Indeed, the combined sum of EPA, DPA, and DHA only constitutes ~30% of the total fatty acids in menhaden oil [37]. To attain the same amount of these fatty acids in purified form, one would only need to consume 1.25, 2.5, 5, 10, or 20 g/day for the 6.25%, 12.5%, 25%, 50%, and 100% FO diets, respectively, to consume the same amount of these fatty acids. Most clinical trials use between 1.25 and 2.5 g of ω-3 LC-PUFAs (combined EPA and DHA), making these two diets particularly clinically relevant.

Mice, unlike humans, efficiently synthesize LC-PUFAs from their respective essential fatty acids. Thus, although the control diet (0% FO) contained no appreciable amounts of AA, EPA, DPA, or DHA (Table 2A), mice synthesized these fatty acids de novo from LA and LNA (linolenic acid), as shown by their presence in RBC membranes (Table 2B). The total PL content (sum of PC, PE, PS, and PI) was unchanged in any of the experimental groups (Figure 6). As such, increasing the concentration of ω-3 LC-PUFAs necessitates a decrease in other fatty acid classes since fatty acids are primarily esterified to glycerol in the form of phospholipids with lesser amounts of sphingolipids, neutral lipids (triglycerides), and free fatty acids. In the high-dose FO groups, ω-3 LC-PUFAs replaced ω-6 LC-PUFAs (Figure 3), resulting in increased fatty acid desaturation and ω-3/ω-6 ratios. These fatty acids can be enzymatically (lipoxygenase; LOX) or spontaneously (peroxidation) modified to bioactive lipids, which are known as oxylipins. Oxylipins derived from ω-6 LC-PUFAs are pro-inflammatory, whereas ω-3-derived oxylipins are pro-resolving [38]. Thus, increased levels of ω-3-derived oxylipins would theoretically be beneficial when transfused into recipients. The substrates of LOX are free fatty acids, whereas peroxidation-based metabolism occurs with both PL-bound and free fatty acids. Thus, oxylipins derived from different LOXs (e.g., 5-LOX, 8-LOX, and 12-LOX, which produce 5-HETE, 8-HETE, and 12-HETE, respectively) reflect enzymatic activity, while oxylipins not associated with a specific LOX (e.g., 9-HETE, 11-HETE) reflect lipid peroxidation and precursor concentration. The levels of oxylipins derived from ω-6 LC-PUFAs (e.g., LA, DGLA, AA) decreased with increasing amounts of dietary FO, and the oxylipins derived from ω-3 LC-PUFAs (EPA, DHA) increased with increased FO (Figure 8). This was true for oxylipins derived both from enzymatic metabolism and peroxidation, suggesting that these changes were primarily determined by differences in substrate levels but not in enzyme activity.

During cold storage, phospholipase activity increases, resulting in increased levels of LPL and fatty acids [39,40]. Several cytosolic phospholipases have substrate specificity for PLs containing LA and AA (e.g., cytosolic Group IV E and F (cPLA2)) [41]. Since the RBC membranes of mice consuming high-dose FO have marked reductions in LA and AA, we hypothesized that RBCs with lower levels of LA and AA would have lower LPL and FFA levels after cold storage. However, we did not observe any differences in LPL or FFA levels in RBCs from mice consuming high-dose FO relative to lower doses of FO after cold storage (Figure 7 and Figure 8). Nonetheless, we did observe significant increases in LPS and LPI levels when comparing stored RBCs to fresh RBCs; LPC and LPE were unchanged after storage (Supplementary Figure S2). Increased levels of LPS and LPI, but not LPC, suggest increased activity of lipases with anionic substrate affinity (i.e., negatively charged phospholipids at physiological pH). Indeed, increased levels after storage of several fatty acids (Supplementary Figure S3) are consistent with increased phospholipase activity previously reported by other investigators.

The average in vivo lifespan of murine RBCs is 40–55 days [25]. During erythropoiesis in the bone marrow and RBC maturation in the circulation, they lose their nucleus and other organelles. As such, mature RBCs cannot synthesize new proteins. As RBCs age in vivo, they accumulate oxidative damage to their membrane, which can be partially rescued by vesiculation [42,43]. Sizable loss of RBC membrane surface area results in decreased deformability [44], splenic retention, and terminal removal from circulation by red pulp macrophages [43,45]. If increased membrane lipid desaturation is associated with increased lipid peroxidation, then one would hypothesize decreases in RBC deformability, lifespan in vivo, and PTR following transfusion. Consistent with this hypothesis, high-dose FO increased MDA in fresh blood units (Figure 5A), decreased deformability (Figure 9A), filterability (Figure 10A), and RBC lifespan in vivo (Figure 4). Nonetheless, no differences in hematocrit or RBC number were seen in mice consuming high dose FO, suggestive of compensatory hematopoiesis (Table 3).

RBCs from C57BL/6J mice fed low-dose FO diets (6.25–25% FO) had significantly improved fresh RBC filterability relative to controls (Figure 10A). Although no improvement in RBC elongation was observed by LORRCA (Figure 9A), microsphiltration is a more functionally relevant measure of deformability, analogous to splenic sequestration, as the RBCs must change shape to filter through the metal beads, similar to the requirement of squeezing through interendothelial slits (IES) in the spleen. IES are very narrow and are the primary site retention for senescent RBCs due to increased rigidity from repeated vesiculation, loss of cell surface area, and resultant shape change. Additionally, diminished aquaporin activity due to ATP depletion, altered cytoskeletal protein structure, and PS externalization contribute to the sequestration of senescent RBCs. Indeed, RBC lifespan in vivo is inversely correlated to microsphiltration retention (Figure 13B), and the relationship between RBC elongation as determined by LORRCA almost reached statistical significance (*p* = 0.08; Figure 13A). The relationship between RBC lifespan and microshiltration remained statistically significant upon performing the same analysis excluding the data point from the 100% FO group (R^2^ = 0.93, *p* = 0.008); however, the relationship with LORRCA was not significant (R^2^ = 0.04, *p* = 0.73).

Although low-dose FO did not reduce lipid peroxidation (i.e., MDA) in fresh RBCs relative to control, unlike high dose FO, it also did not increase lipid peroxidation. Improved filterability of fresh RBCs did not improve fresh RBC PTR (Figure 11A), likely because the PTR at baseline was already optimal (~100%) with no opportunity for further enhancement. However, after 12 days of cold storage, MDA levels were further increased in the high-dose FO groups (50% and 100% FO; Figure 5B), with reduced deformability (Figure 9B), filterability (Figure 10B), and PTR (Figure 11B) relative to fresh blood units. Conversely, low-dose FO reduced MDA, suggesting that it provided a protective antioxidant effect (Figure 5B). However, reduced lipid peroxidation in stored RBCs conferred by low-dose FO did not improve deformability (Figure 9B), filterability (Figure 10B), or PTR (Figure 11B) relative to controls. Deformability and filterability of 12-day stored RBCs significantly correlated with PTR, explaining 87.7% and 94.3% of the variance in PTR, respectively (Figure 12A,B); this is consistent with the hypothesis that RBCs are cleared in vivo by splenic sequestration due to decreased deformability. The relationship between PTR and microshiltration remained statistically significant upon performing the same analysis excluding the data point from the 100% FO group (R^2^ = 0.86, *p* = 0.02); however, the relationship with LORRCA was no longer statistically significant (R^2^ = 0.57, *p* = 0.14). 

Overall, high-dose dietary FO was deleterious to RBC deformability, in vivo lifespan, and susceptibility to oxidative damage. With respect to RBC membrane composition, several mechanisms can explain these observations. First, RBCs from mice fed high-dose FO had large increases in the relative composition of LC-PUFAs in their membrane. Increases in the number of bis-allylic positions provide more sites for hydrogen abstraction to occur, thereby enhancing the propensity for lipid peroxidation. The high-FO diets also had decreased levels of LA (Table 2A), an ω-6 LC-PUFA, which resulted in lower incorporation of LA into the RBC membrane (Table 2B).

L-carnitine has important biochemical functions; one of its most well-documented roles is the transport of long-chain fatty acids across the inner mitochondrial membrane [46]. However, since most mature RBCs lack mitochondria, carnitine is hypothesized to have an alternative function in RBCs, providing an indirect reservoir of activated fatty acids (Acyl-CoA) as substrates for lysophospholipid acyltransferase. Thus, oxidized fatty acids in the phospholipid bilayer can be enzymatically removed by phospholipases and subsequently replaced with new fatty acids (i.e., the Lands’ Cycle). Although the intracellular Acyl-CoA pool is small and requires ATP to synthesize new Acyl-CoA molecules, the acylcarnitine pool is significantly larger and can be transesterified to Acyl-CoA by an ATP-independent pathway [47]. Additionally, free and esterified carnitine have antioxidant properties [48]. Since lipid peroxidation is a hallmark of the RBC storage lesion, previous studies hypothesized that carnitine supplementation could attenuate these negative effects by reducing lipid peroxidation, increasing antioxidant capacity, and replacing oxidized lipids via the Lands’ Cycle [49]. Our RBC metabolomics results are consistent with this hypothesis. In both fresh and stored RBCs, we observed a biphasic response in free and esterified carnitine species, with concentrations increasing from 0% FO to 12.5% FO before sharply decreasing down to the 100% FO group (Figure 14). The stored RBC acylcarnitine data are consistent with markers of lipid peroxidation, in which MDA decreased from the 0% FO to the 12.5% FO diet group, followed by an increase through the 100% FO group (Figure 5B). These results can be explained by the hypothesis that acylcarnitines act as a reservoir of “normal” fatty acids to replace the oxidized ones in the RBC membrane. We hypothesize that low levels of acylcarnitines in RBCs from the mice fed higher FO doses are caused by depletion of the acylcarnitine reservoir, consistent with the higher measured levels of oxidative stress.

Although increasing the concentrations of LC-PUFAs relative to saturated and less saturated fatty acids in the RBC membrane improves membrane fluidity and deformability, increases in lipid peroxidation can counteract this benefit and actually reduce deformability, as was observed in mice fed high-dose dietary FO. Additionally, the FO dose at which decreased RBC quality is observed may exhibit biological variability based on basal rates of ROS production; thus, individuals with elevated basal ROS exposure (e.g., patients with sickle cell disease) could tolerate smaller amounts of dietary FO. In a recent study, the addition of antioxidants to RBCs, such as ascorbic acid and uric acid, protected the RBCs from oxidative stress during cold storage by upregulating their antioxidant capacity [50]. Based on the results presented herein, we hypothesize that co-administering antioxidants along with dietary FO may attenuate the increased lipid peroxidation and improve RBC deformability. This has the potential to provide a clinical benefit to both the supplemented donor and the recipient of those donated RBCs (in the context of transfusion). The RBC in vivo lifespan (Figure 4) was similar for mice fed diets containing pharmacologically relevant doses of FO (0–12.5%) before decreasing at higher doses. Additionally, mice fed up with 12.5% FO exhibited improved RBC filterability, with subsequent doses decreasing deformability (Figure 10A). This suggests a potential benefit for co-supplementation with both FO and antioxidants, as any deleterious effects associated with increased oxidative stress could be mitigated by increasing the antioxidant capabilities of the RBCs. The improvement in RBC deformability could benefit endurance athletes and patients with conditions resulting from reduced perfusion, such as peripheral vascular disease. Along with improved donor RBC quality, we also hypothesize improved stored RBC quality and, ultimately, improved clinical outcomes following RBC transfusion.

## Figures and Tables

**Table 1:** The fat sources in each FO diet.

Percent Fish Oil						
Fat Source	0%	6.25%	12.5	25%	50%	100%
	Concentration in Diet (g/kg)					
Milkfat	47.3	44.3	41.3	35.4	23.6	0.0
Olive Oil	13.5	12.7	11.8	10.1	6.8	0.0
Safflower Oil	60.8	57.0	53.2	45.6	30.4	0.0
Canola Oil	13.5	12.7	11.8	10.1	6.8	0.0
Menhaden Oil	0.0	8.4	16.9	33.8	67.5	135.0

**Table 2:** Fatty acid profiles of diets (A) and RBCs (B). Groups not sharing common letters differ statistically by one-way ANOVA with Tukey’s multiple comparisons follow-up test (*p* < 0.05).

A		Percent Fish Oil					
Total Fatty Acid		0%	6.25%	12.5%	25%	50%	100%
		Concentration in Diet (µmol/g)					
C12	Lauric Acid	10.3 ± 2.8 ^A^	8.2 ± 1.7 ^A^	9.9 ± 4.3 ^A^	4.8 ± 2.1 ^AB^	4.9 ± 0.8 ^B^	1.1 ± 0.1 ^B^
C14	Myristic Acid	18.2 ± 3	16.2 ± 4.2	18.9 ± 5.6	14.9 ± 2	25.1 ± 8	26.3 ± 1.2
C16	Palmitic Acid	154.3 ± 10.4 ^A^	129.6 ± 2 ^BC^	115.2 ± 10.8 ^B^	127.0 ± 3.9 ^BC^	134.3 ± 4.7 ^C^	125.7 ± 3.7 ^BC^
C16:1	Palmitoleic Acid	0.7 ± 0.1 ^A^	1.2 ± 0.3 ^A^	2.0 ± 0.7 ^A^	2.4 ± 0.2 ^A^	6.6 ± 3.6 ^B^	0.7 ± 0.1 ^A^
C18	Stearic Acid	62.6 ± 2.5 ^A^	50.6 ± 4.6 ^AB^	41.3 ± 9.7 ^B^	53.8 ± 1.6 ^AB^	48.2 ± 6.1 ^B^	46.6 ± 1.3 ^B^
C18:1	Oleic Acid	85.3 ± 10.3 ^A^	72.7 ± 17.4 ^A^	70.8 ± 17.3 ^A^	56.5 ± 4.1 ^A^	57.2 ± 20.7 ^A^	14.8 ± 1 ^B^
C18:2	Linoleic Acid (LA)	128.5 ± 14.1 ^A^	107.9 ± 22.7 ^AB^	106.4 ± 23.9 ^AB^	83.4 ± 6.8 ^AB^	75.8 ± 25.1 ^B^	11.5 ± 0.5 ^C^
C18:3	Alpha-Linoleic Acid (ALA)	3.9 ± 0.4	3.6 ± 0.4	3.8 ± 0.5	4.0 ± 0.2	5.5 ± 1.6	4.9 ± 0.5
C20	Arachidic acid	1.3 ± 0.1 ^A^	1.2 ± 0.2 ^A^	1.1 ± 0.1 ^AB^	1.2 ± 0.1 ^A^	1.2 ± 0.2 ^A^	0.8 ± 0 ^B^
C20:1	Eicosenoic Acid	0.8 ± 0.1 ^A^	0.9 ± 0.1 ^A^	0.9 ± 0.1 ^A^	1.0 ± 0.1 ^A^	1.7 ± 0.5 ^B^	1.8 ± 0.1 ^B^
C20:4	Arachidonic Acid (AA)	0.2 ± 0 ^A^	0.3 ± 0.1 ^A^	0.5 ± 0.1 ^A^	0.8 ± 0 ^A^	1.7 ± 0.7 ^B^	2.2 ± 0.3 ^B^
C20:5	Eicosapentaenoic Acid (EPA)	0.1 ± 0 ^A^	2.1 ± 0.4 ^A^	4.4 ± 0.9 ^A^	8.3 ± 0.5 ^A^	22.0 ± 8.3 ^B^	28.5 ± 2.6 ^B^
C22:1	Erucic acid	1.0 ± 0.1	1.3 ± 0.1	1.2 ± 0.3	1.1 ± 0.1	1.0 ± 0.3	0.9 ± 0.2
C22:5	Docosapentaenoic Acid (DPA)	0.1 ± 0 ^A^	0.3 ± 0.1 ^A^	0.6 ± 0.1 ^A^	1.0 ± 0.1 ^A^	2.9 ± 1 ^B^	3.6 ± 0.4 ^B^
C22:6	Docosahexaenoic Acid (DHA)	0.3 ± 0 ^A^	1.7 ± 0.3 ^A^	3.3 ± 0.7 ^A^	6.4 ± 0.7 ^A^	15.6 ± 6 ^B^	20.1 ± 2.1 ^B^
**B**		**Percent Fish Oil**					
**Total Fatty Acid**		**0%**	**6.25%**	**12.5%**	**25%**	**50%**	**100%**
		**Concentration in Fresh RBCs (µmol/L)**					
C12	Lauric Acid	6.0	8.2	13.0	9.7	4.2	7.5
C14	Myristic Acid	59.3	105.8	118.1	107.0	122.8	125.4
C16	Palmitic Acid	6134.9	9189.9	9355.5	8425.4	7450.5	6640.1
C16:1	Palmitoleic Acid	77.8	112.7	124.9	120.3	160.3	203.3
C18	Stearic Acid	2446.2	3143.7	3247.2	2777.9	2221.2	2137.9
C18:1	Oleic Acid	1787.3	2686.0	2779.4	2235.7	1662.9	1215.4
C18:2	Linoleic Acid (LA)	2108.0	3309.4	3588.2	2662.9	1643.7	321.6
C18:3	Alpha-Linoleic Acid (ALA)	12.4	16.8	22.9	17.7	21.0	16.7
C20	Arachidic acid	40.7	60.9	58.9	58.0	41.6	35.9
C20:1	Eicosenoic Acid	35.7	50.4	48.6	33.0	33.0	20.2
C20:4	Arachidonic Acid (AA)	2539.9	2601.8	2134.7	1457.8	855.5	809.8
C20:5	Eicosapentaenoic Acid (EPA)	10.4	241.8	510.9	800.6	1036.9	1187.3
C22:1	Erucic acid	26.5	23.1	26.5	24.0	23.5	23.7
C22:5	Docosapentaenoic Acid (DPA)	47.0	193.3	265.5	293.9	305.0	253.4
C22:6	Docosahexaenoic Acid (DHA)	638.0	1380.5	1595.3	1574.0	1354.1	1299.3

**Table 3:** Hematological profiles of blood pooled from mice consuming various diets. Groups that do not share common letters statistically differ from each other by one-way ANOVA with Tukey’s multiple comparisons follow-up test (*p* < 0.05).

CBC Measurement	Units	Percent Fish Oil					
		0%	6.25%	12.5%	25%	50%	100%
Hematocrit	(%)	36.2% ± 2.0%	35.4% ± 3.8%	33.7% ± 2.1%	37.5% ± 1.3%	37.2% ± 2.7%	36.1% ± 0.9%
RBC Count	(10^12^/L)	9.0 ± 0.6	8.4 ± 0.7	8.2 ± 0.5	8.9 ± 0.3	8.9 ± 0.5	8.7 ± 0.2
Hemoglobin	(g/dL)	11.5 ± 0.6	11.2 ± 1.2	10.4 ± 0.7	11.6 ± 0.5	11.7 ± 0.7	11.0 ± 0.3
RBC Distribution Width	(%)	18.5% ± 1.5%	17.9% ± 1.0%	18.2% ± 1.6%	17.4% ± 0.4%	18.3% ± 0.7%	18.8% ± 0.7%
Mean Corpuscular Volume (MCV)	(fL)	52.5 ± 4.5	51.2 ± 4.7	48.7 ± 1.4	50.1 ± 1.1	50.7 ± 3.0	50.1 ± 2.0

## Data Availability

Data are available upon reasonable request.

## Supplementary Materials

The following supporting information can be downloaded at: https://www.mdpi.com/article/10.3390/nu15204456/s1, Figure S1: Ratio of stored PLs to fresh PLs in RBCs. Differences between fresh and stored blood were assessed by comparing the fold change to 1 using a *t*-test (n = 4). Figure S2: Ratio of stored LPLs to fresh LPLs in RBCs. Differences between fresh and stored blood were assessed by comparing the fold change to 1 using a *t*-test (n = 4). * *p* < 0.05; ** *p* < 0.01. Figure S3: Ratio of stored FFAs and oxylipins to fresh FFAs and oxylipins. Differences between fresh and stored blood were assessed by comparing the fold change to 1 using a *t*-test (n = 4). * *p* < 0.05.
