# Supplementary figures and images for: Low-Dose Dietary Fish Oil Improves RBC Deformability without Improving Post-Transfusion Recovery in Mice
Source: Nutrients. 2023 Oct 20;15(20):4456. doi: 10.3390/nu15204456 (PMC10610231; doi:10.3390/nu15204456)

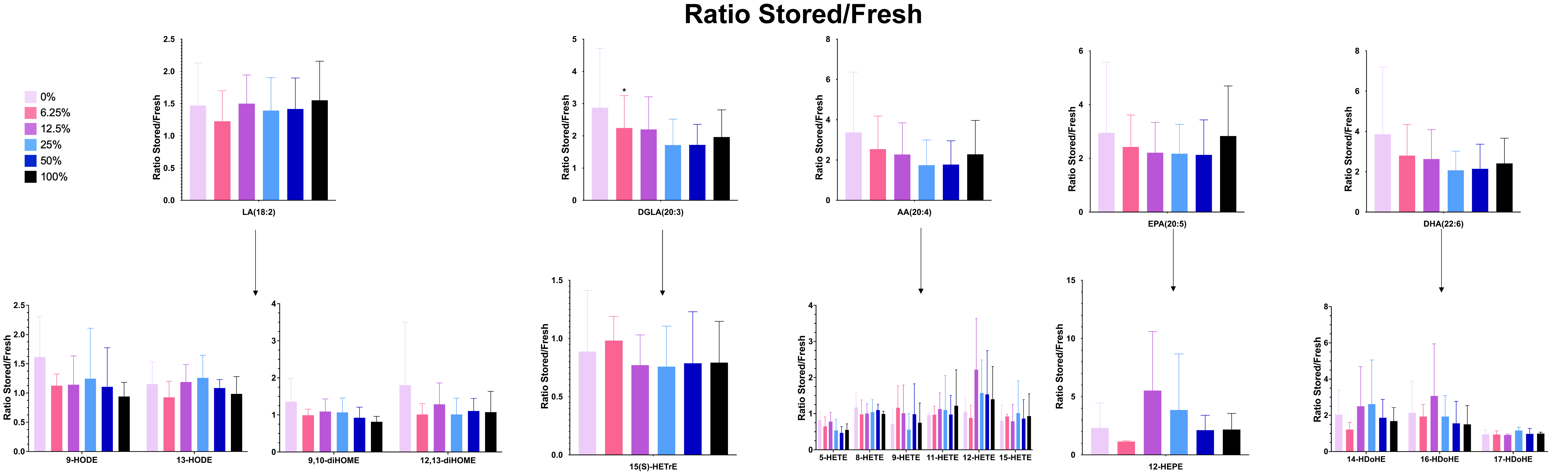

Supplement: Supplementary file 1 [file nutrients-15-04456-s001.zip › FO Supplemental F3.tif]
